# Impact of a poison control center on the length of hospital stay of poisoned patients: retrospective cohort

**DOI:** 10.1590/S1516-31802011000100005

**Published:** 2011-01-06

**Authors:** Taís Freire Galvão, Marcus Tolentino Silva, Carolina Dalene Silva, Adriana Melo Barotto, Izabela Lucchese Gavioli, Fábio Bucaretchi, Álvaro Nagib Atallah

**Affiliations:** I Pharmacist, Amazonas Poison Control Center, Getúlio Vargas University Hospital, Universidade Federal do Amazonas (UFAM), Manaus, Amazonas, Brazil.; II Pharmacist, Department of Science and Technology, Secretariat of Science and Technology and Strategic Inputs, Ministry of Health, Brasília, Distrito Federal, Brazil.; III Medical student, Amazonas Poison Control Center, Getúlio Vargas University Hospital, Universidade Federal do Amazonas (UFAM), Manaus, Amazonas, Brazil.; IV MD. Endocrinologist and clinical coordinator of Santa Catarina Poison Control Center, University Hospital, Universidade Federal de Santa Catarina (UFSC), Florianópolis, Santa Catarina, Brazil.; V MD. Rheumatologist, Rio Grande do Sul Poison Control Center, Fundação Estadual de Produção e Pesquisa em Saúde (FEPPS), State Health Department, Porto Alegre, Rio Grande do Sul, Brazil.; VI MD, PhD. Assistant professor and coordinator of Campinas Poison Control Center, Hospital das Clínicas, Universidade Estadual de Campinas (Unicamp), Campinas, São Paulo, Brazil.; VII MD, MSc, PhD. Nephrologist. Director of the Brazilian Cochrane Center and titular professor of Evidence-Based Medicine and Emergency Medicine, Universidade Federal de São Paulo — Escola Paulista de Medicina (Unifesp-EPM), São Paulo, Brazil.

**Keywords:** Poison Control Centers, Length of stay, Poisoning, Hospitalization, Health services research, Centros de Controle de Intoxicações, Tempo de internação, Envenenamento, Hospitalização, Pesquisa sobre serviços de saúde

## Abstract

**CONTEXT AND OBJECTIVE:**

Poison control centers play an essential role in caring for poisoned patients, albeit without secure funding for their activities. The aim here was to investigate differences in length of hospital stay among poisoned patients, between those who received remote assistance from a poison control center and those who did not.

**DESIGN AND SETTING:**

A retrospective cohort including all poisoned patients hospitalized at an emergency service in Manaus between 2005 and 2007 was set up, and the local poison control center database was checked to see whether they received such assistance.

**METHODS:**

Patients presenting a known toxic agent, with less than 12 hours since exposure and without severe comorbidities, were selected. Their severity of poisoning was evaluated by two independent reviewers and divergences were resolved by another reviewer.

**RESULTS:**

One hundred and ninety-eight patients were included. Those who received remote assistance from a poison control center stayed in hospital on average for 3.43 days less than those without poison control center assistance (95% confidence interval, CI: -6.10 to -0.77). Severity was assessed in the cases of 90 patients: there was no statistical difference in severity between the patients with and without poison control center assistance (P > 0.5).

**CONCLUSION:**

Patients with remote assistance from a poison control center had a shorter length of stay then patients without this aid. The poison control center may have reduced the length of stay of the poisoned patients.

## RESUMO

**CONTEXTO E OBJETIVO:**

Centros de informações toxicológicas prestam papel essencial na assistência a pacientes intoxicados, sem contar com financiamento seguro de suas atividades. O objetivo foi verificar a diferença no tempo de internação dos pacientes intoxicados que receberam atenção remota de um centro de informações toxicológicas em comparação aos que não receberam.

**TIPO DE ESTUDO E LOCAL:**

Foi organizado um estudo de coorte retrospectivo incluindo todos pacientes intoxicados hospitalizados em um prontosocorro de Manaus de 2005 a 2007 e checado na base de dados do centro de informações toxicológicas local se receberam esta assistência.

**MÉTODOS:**

Foram selecionados pacientes com agente tóxico conhecido, tempo de exposição inferior a 12 horas e sem comorbidades graves para avaliação da gravidade por dois revisores independentes e divergências resolvidas por outro revisor.

**RESULTADOS:**

Foram incluídos 198 pacientes; aqueles com auxílio remoto do centro de informações toxicológicas ficaram em média 3,43 dias a menos (-6,10 a -0,77 IC 95%) internados quando comparados a nenhum auxílio do centro de informações toxicológicas (CIT). Noventa pacientes tiveram gravidade avaliada; não houve diferença estatística na gravidade entre os pacientes com ou sem assistência do CIT (P > 0,5).

**CONCLUSÃO:**

Pacientes com assistência remota do CIT tiveram tempo de internação inferior a pacientes sem este auxílio. O CIT pode ter reduzido o tempo de internação dos pacientes intoxicados.

## INTRODUCTION

Development of technologies has stimulated proliferation of different chemical substances. More than 60 million chemical substances have been registered in the world, and around 40 million of them are commercially available.^1^ Exposure of the population to these substances may result in poisoning,^2,3^ which is an important cause of morbidity and mortality worldwide.^4^

Acquisition of data to identify such substances and classify them according to their pharmacological nature, and organization of information that is pertinent for preventing, diagnosing and treating cases of poisoning, are strategic actions for minimizing the potential damage caused by exposure to chemical substances. Such efforts began in the 1940s in Europe^5^ and in the 1970s in Brazil,^6^ in the form of services organized under the following names: Poison Control Centers, Poisoning Information Centers, Antivenin Information Centers and Toxicological Information and Assistance Centers, among others. Since then, these centers have made it possible to provide continuous guidance and advice on how to deal with toxic exposure to chemical substances and animal bites. They are accessible by telephone, 24 hours a day throughout the year, including attendance in person for some services.

Poison control centers count on voluntary work performed by professionals, or work by professionals who have been recruited from other services to develop these centers' activities. Despite the lack of secure and organized sources of treatment for poisoning, such centers have constructed and refined a large proportion of the field of clinical toxicology. Today, evidence-based guidelines are available, coming from clinical studies and, especially, epidemiological data that has been collected in an organized manner from attendance provided by these centers.^7^

Despite all the benefits that these centers may bring for society, the funding for these structures is unstable. In Brazil, there are 36 active centers,^8^ and they are not formally part of the public national health system (Sistema Único de Saúde, SUS). This requires a sensitive attitude from all administrators at the institutions to which these services belong, in order to enable investment in and maintenance of these services. Other countries share similar scenarios, in which the centers are funded through chains of mechanisms that include local healthcare departments, university departments and hospitals.^9^ These do not provide security of maintenance for these services.

To show the importance of poison control centers, several studies have been conducted, especially in the field of economic evaluation.^10-18^ These studies have indicated that the centers' activities have led to savings in healthcare resources, and that if such services are absent, the emergency services are overloaded and have greater expenditure.

In Brazil, there is still a lack of studies evaluating these centers' services, especially in relation to their impact on attendance provision. Given that around 70% of the Brazilian population depends on SUS for healthcare assistance^19,20^ (a situation that differs from healthcare systems in other countries), national studies investigating the impact of such centers on healthcare would provide support for policies relating to funding and consolidation of these services.

Another peculiarity that needs to be borne in mind is that the Brazilian poison control centers mainly attend to requests coming from healthcare units. This differs from what is seen in developed countries, in which most cases are managed at the locality of occurrence. In the State of Amazonas, while 83% of the attendance provided by the center in 2007 was in relation to occurrences in homes, 65% of the requests came from healthcare services.^21^ In the same year, 63% of the calls to the center in Rio Grande do Sul came from healthcare establishments.^22^ On the other hand, in the United States, 72.5% of the calls to centers in 2009 came from homes.^23^

Conducting investigations to evaluate patient care improvement indicators at SUS healthcare units is one strategy for assessing the potential of the Brazilian poison control centers. In addition to enabling support for decision-making in this field, this may stimulate further investigations within academic circles.

One practical way of evaluating the improvement in care for SUS patients that these centers provide is to examine the length of hospitalization among such patients. In New Jersey, United States, a study showed shorter hospitalization among patients who had received care from the local poison control center.^24^ In association with this measurement, evaluation of the severity of the patients' conditions would help in investigating the effect.

## OBJECTIVE

The present study was designed with the aim of answering the question: “Is there a difference in the length of hospitalization among poisoned patients between those who received help from a center and those who did not receive such help?” Our hypothesis was that poison control center assistance might reduce the length of hospital stay among poisoned patients and thus represent an effective strategy for achieving quality assistance, along with cost containment, within clinical toxicology care.

## METHODS

A retrospective cohort was organized, in which patients hospitalized due to poisoning at the “28 de agosto” Hospital and Emergency Service were retrospectively observed to investigate outcomes and whether remote assistance had been provided by a poison control center, taking such help to be a protection factor.

The hospital and emergency service studied here is a public general hospital run by the state authorities. It is registered in the National Register of Healthcare Establishments for provision of outpatient and hospital services of medium complexity destined for the adult population, and it is a referral emergency service for the state. It has a surgical center with six rooms, 75 observation beds, 218 ward beds for the specialties of general surgery, orthopedics/traumatology, plastic surgery, cardiology, general clinical medicine and nephrology/urology, and 20 beds in an intensive care unit for adults.^25^

The data were gathered between June and October 2008. At this time, the records on patients who had been hospitalized with a primary diagnosis of poisoning, admitted through the emergency service between 2005 and 2007, were retrieved. Subsequently, it was investigated whether these patients had received any assistance from the Amazonas Poison Control Center, through consulting the poisoning record system of the center.

Amazonas Poison Control Center is a service within the Getúlio Vargas University Hospital of the Universidade Federal do Amazonas (UFAM). It specializes in rapidly and succinctly making evidence-based guidance available, regarding procedures for preventing, diagnosing and treating cases of poisoning. Through a continually available telephone service (remote attendance), people seeking help can obtain specific information for managing poisoning caused by a very wide range of toxic agents. After this initial contact, patients start to be monitored by the center's team. Through daily contact (by telephone), the team follows up and collaborates with the evolution of the case.^21^ For hospitalized patients to receive assistance from the poison control center, a professional involved in treating such patients needs to contact the center to receive guidance regarding the best approach to institute and the procedures to avoid.

To evaluate the severity of patients' conditions, patients with the following characteristics at the time of hospitalization were selected: the toxic agent to which the patient was exposed was known in qualitative terms; exposure occurred not more than 12 hours before attendance at the emergency service; and absence of severe comorbidities. The latter were defined as pathological conditions with the potential to corroborate the severity of the general condition or that, in themselves, required treatment.

The study variables were: year, sex, age, entry date, discharge date, length of hospitalization, provision of help from the poison control center, toxic agent, clinical history on admission, evolution, clinical manifestations and severity assessment.

The data on all the patients thus identified were gathered by one of the investigators (CDS), from the hospitalization register that was available at the Medical and Statistical Archiving Service of the emergency service. The length of hospital stay (in days) was calculated from the admission and discharge dates.

Observations regarding the patients who had received remote attendance from the poison control center were obtained independently by another investigator (TFG), by searching for names and dates of attendance in the poison control center's database. This procedure was followed in order to avoid measurement bias.

To analyze the severity of the cases, the medical files of the patients selected were gathered by one investigator (TFG) on a form that had been standardized for this purpose. The forms were sent electronically to two independent evaluators (AMB and ILG), who were physicians at different poison control centers in Brazil with high experience in clinical toxicology. For their assessment, these evaluators did not receive any information on whether remote assistance had been received from the center. Cases presenting classification disagreements were analyzed by a third evaluator who was a physician, university professor with a doctorate and coordinator of a poison control center in the state of São Paulo (FB).

The Poisoning Severity Score,^26^ a score that has been validated for use in classifying the severity of poisoning, was used at this stage. This score was developed through collaborative work involving 14 centers in several countries, including Brazil. The score uses five grades of severity, based on the observation of clinical manifestations: (0) none reported; (1) mild; (2) moderate; (3) severe; and (4) fatal.

The analysis on the severity of the patients' cases, along with comparison of the patients with and without remote assistance from the center were stages inserted into the assessment in order to avoid sampling bias.

The data for this study came from the medical file register and were subject to errors and omissions of information cause by lack of standardization of the medical records, bearing in mind that these records had not been maintained for clinical research.^27,28^ The hospital at which this investigation was performed did not have a fully computerized system for registering the medical files (a situation that was common among other public emergency services in this state), which made the process of locating the records more laborious.

It was decided to study all the patients hospitalized at this emergency service due to poisoning in the years 2005, 2006 and 2007, because it would be practicable to obtain these data, taking into consideration the size of the team involved in the study, the archiving system at this emergency service and the feasibility of retrieving information from the poison control center's database.

The frequencies of the variables “year”, “number of toxic agents” and “severity classification” were obtained. The mean and standard deviation were calculated for the variables “age” and “length of hospitalization”. The kappa index^29^ was calculated to analyze the concordance of the severity classification between the evaluators.

We analyzed comparative statistical differences between the categorical variables through calculations using the chi-square test, and Fisher's exact test when appropriate. The continuous variables were evaluated statistically through calculations using Student's t test, analysis of variance (Anova) and mean difference, when appropriate. In addition, the statistical description of these variables (sum, mean, minimum, maximum, standard deviation and median) was presented. The data were tabulated using the Microsoft Excel® 2003 software and were analyzed using the Epidat 3.1^30^ software.

The study was authorized by the hospital's board (authorization dated March 6, 2008) and by the research ethics committee of Universidade Federal de São Paulo (Unifesp), through decision number 1028/2008.

## RESULTS

Over the three-year period, it was found that 198 patients had been hospitalized due to poisoning seen at the emergency service, and these individuals were included in the study. In total, these patients accounted for 1,568 days of hospitalization. Most of the patients belonged to the group that did not receive assistance from the poison control center and were male (69.7%). The mean age among these patients was 37.54 ± 17.67 years. The mean annual number of hospitalization was 66 ± 10.44. The baseline characteristics of these patients, according to the group to which they belonged, are summarized in **Table 1**.

**Table 1.** Main demographic features of the two study groups

Basic features	PCC remote assistance	No PCC assistance	Total
Patients (n; %)	36; 35.5	162; 64.5	198; 100.0
Age (mean ± SD)*	29.44 ± 12.22	39.33 ± 18.21	37.54 ± 17.67
Gender (n)†			
male	21	117	138
female	15	45	60
Year of admission (n)†			
2005	13	60	73
2006	10	44	54
2007	13	58	71

* Difference between groups was statistically significant (P < 0.001).

† Difference between groups was statistically non-significant (P > 0.05).

PCC = poison control center; SD = standard deviation.

**Figure 1.**
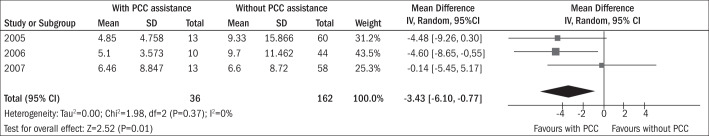
Mean length of hospital stay and mean difference in length of stay between groups, i.e. with and without poison control center (PCC) assistance.

It was observed that the hospitalized patients who had received aid from the poison control center remained hospitalized for a mean of 5.50 ± 6.20 days, while the patients without any aid from the center remained hospitalized for a mean of 8.46 ± 12.50 days. The mean difference between the two groups was -3.43 days (95% confidence interval, CI: -6.10 to -0.77), thus revealing that the patients with remote assistance from the poison control center remained hospitalized for shorter periods than did patients who did not receive such help (**Figure 1**).

**Figure 2.**
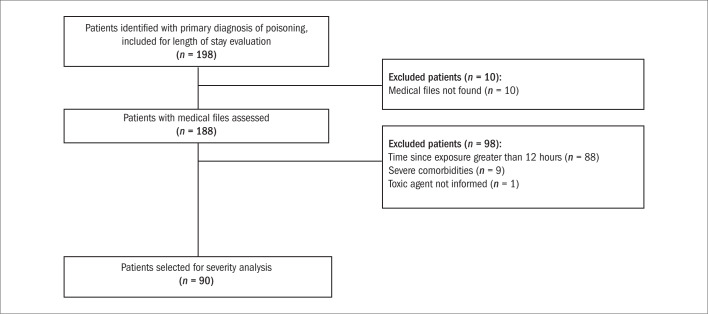
Flow chart of patients selected for severity analysis, with the poisoning severity score.

Among the patients selected for severity analysis (**Figure 2**), there was no difference in gender distribution; the mean age was 30.46 ± 14.24 years; and 35.5% of the patients received assistance from the poison control center. The patients who had received assistance from the poison control center remained hospitalized for a mean of 4.66 ± 3.90 days, while patients without aid from the center remained hospitalized for a mean of 5.66 ± 6.88 days. There were no statistically significant differences in length of hospital stay among those patients (P > 0.05).

The severity assessments made by the independent evaluators presented significant concordance, with a weighted kappa result of 0.68 (95% CI: 0.57 to 0.79), using quadratic weights. There were 34 discordant cases (**Table 2**), and these patients presented statistically significant differences in relation to the other patients, for the variables of age, sex, complications and hospitalization in the intensive care unit (P < 0.05).

**Table 2.** Severity classification distributed according to reviewer

Reviewer 2							
		None (0)	Minor (1)	Moderate (2)	Severe (3)	Fatal (4)	Total
Reviewer 1	**None (0)**	0	2	0	0	0	**2**
	**Minor (1)**	0	**10**	8	0	0	**18**
	**Moderate (2)**	0	7	**30**	**15**	0	**52**
	**Severe (3)**	0	0	2	**14**	0	**16**
	**Fatal (4)**	0	0	0	0	2	**2**
	**Total**	0	**19**	20	29	2	**90**

Note: concordances are in bold type.

The severity classification, after analysis by a third reviewer only in the cases with conflicts in the assessment, revealed that most of the patients were classified as presenting moderate poisoning (58.9%) or severe poisoning (20.0%). **Table 3** shows the final evaluation. The concordance between each evaluator and the consensus evaluator was calculated, and this varied from moderate (0.42; 95% CI: 0.22 to 0.61) to substantial (0.72; 95% CI: 0.5 to 0.94). The concordance was greater among evaluators with greater contact with patients; these evaluators' poison control centers were located in hospitals with an emergency service, with attendance in person provided in addition to telephone contact.

**Table 3.** Final severity evaluation

Severity evaluation	N	%
None (0)	2	2.2
Minor (1)	15	16.7
Mild (2)	53	58.9
Severe (3)	18	20.0
Fatal (4)	2	2.2

The groups were comparable from the point of view of severity, which thus minimized the sampling bias. The same was observed among patients with divergences in severity assessment and patients with concordance in this evaluation.

**Figure 3.**
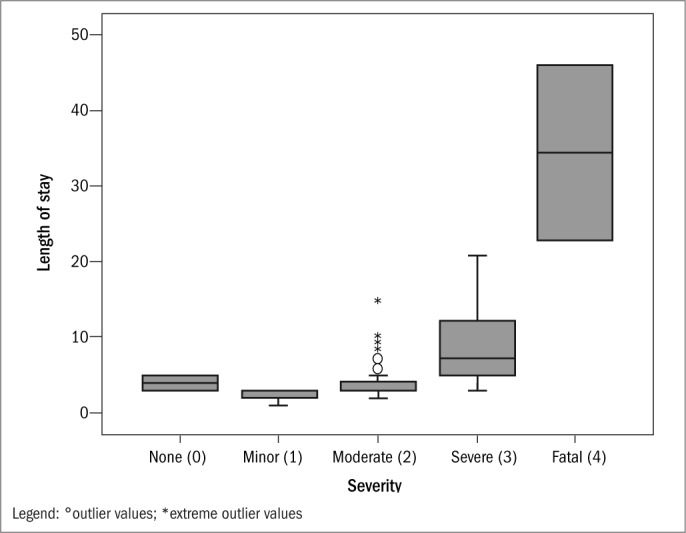
Box plot of patients' severity, distributed according to mean length of hospital stay.

The hypothesis that there would be an association between severity and length of hospitalization was evaluated and found to be statistically significant (P < 0.0001), using Anova. According to the data obtained, the greater the severity of the case was, the greater the length of hospitalization also was. Patients with mild poisoning remained hospitalized for a mean of 2.53 ± 0.64 days, while patients with severe conditions remained hospitalized for 8.72 ± 5.25 days (**Figure 3**).

## DISCUSSION

Patients who received remote aid from the poison control center during the study period remained hospitalized for shorter times on average, compared with patients who did not receive such assistance, during the same period. A similar result was found in the study by Vassilev and Marcus,^24^ who analyzed 31,063 records and noted that patients with assistance from the poison control center remained hospitalized for a mean of 3.95 ± 6.16, while patients without such assistance remained hospitalized for 6.94 ± 7.83, on average.

The result found may represent an important way of containing costs within tertiary healthcare, which has been presenting constantly rising expenditure. In Brazil, hospital care consumes around 50% of the annual resources allocated to SUS. In 2007, this budget was around 27 billion dollars.^31^

Furthermore, in Brazil, the number of beds available is lower than what is needed. Greater turnover of bed use may help to make the provision adequate. According to the Brazilian Institute for Geography and Statistics, 443,510 hospital beds were available in 2005, which is equivalent to 2.4 beds/1,000 inhabitants.^32^ This figure was lower than the means seen in the United States, Canada and Europe region, which were 3.1, 3.4 and 6.3 beds/1,000 inhabitants, respectively.^33^ In general, greater concentrations of beds are associated with higher purchasing power among the population and greater demand for specialized services, which are attractive for private investments.^34^

Another point that deserves attention was that only 18.2% of the study population had had remote help from the poison control center. Similar data were found from an American study, in which aid from a local center was requested in 19% of the 2,494 cases attended at an emergency service.^35^ These data confirm that these specialized support services were underused. The potential benefits that the center provides within the field of clinical toxicology are restricted by lack of investment in their human and material resources, and by lack of dissemination to the public, especially among healthcare professionals.

The Ministry of Health Ordinance no. 298/2010^36^ provides for drawing up guidelines for actions within SUS relating to care and surveillance in the field of clinical toxicology. This brings the real possibility of recognition and integration of the centers within SUS, through national guidelines, in contrast to the current situation, which is dependent on local voluntary action.

The patients included in this study presented homogenous distribution regarding their baseline characteristics, with the exception of age, which was statistically lower among the group that had assistance from the centers. This may have been due to the sample size, thus indicating the need to expand the sample in future studies, in order to avoid random effects.

One important observation that would give greater validity to the result would be to investigate the severity of poisoning among all the patients (198 subjects included), in order to evaluate whether shorter hospitalization was associated with prognoses that were more favorable. However, the occurrence of confounding factors, such as exposure for more than 12 hours, presence of comorbidities and not knowing what the toxicological agent was (in cases of attempted suicide or when the patient is unconscious it is difficult to ascertain the poisoning agent), prevented assessment of the severity of poisoning in relation to all the patients.

There was substantial agreement in the severity assessments between the two professionals working in different poison control centers. This shows that it is feasible to standardize this tool among the different centers in Brazil. The discordant cases presented statistically significant differences in the variables of age, sex, complications and hospitalization in an intensive care unit, in relation to the patients with concordant analyses. Thus, further investigation is required in order to find out what the real influence of these factors is on the decision-making process. The findings point towards the need for training to harmonize the application of the scale.

Consensus analysis for the cases of conflict showed that there was greater concordance among the professionals working in poison control centers that provided attendance both by telephone and in person. This indicates that greater sensitivity may be provided through direct contact with poisoned patients in emergency situations. The World Health Organization recommends that poison control centers should be set up in hospitals in order to facilitate updating and expansion of the data on diagnosing and treating local cases, and to favor detailed follow-up for patients and stimulate clinical research in this field.^37^ It is difficult to conduct experimental studies within clinical toxicology because of ethical, legal and political issues. Hence, this reinforces the importance of maintaining and expanding these services in emergency treatment units.^38^

The final severity assessment showed that most of the patients presented moderate or severe conditions that were conducive to treatment within hospital settings. Nonetheless, there were also considerable numbers of patients classified as presenting mild poisoning, with mild and transitory symptoms that resolved spontaneously.^26^ The demand for services from such patients could be met outside of healthcare units, through guidance regarding simple measures given by the team at a poison control center, thereby avoiding overload on the system and even avoiding personal inconvenience for such patients.^16^

The observation that there was homogeneity between the groups with regard to severity is important for validation of the study data, thereby reducing the sampling bias. Another finding from the present study that might have been expected was the statistically significant association between severity and length of hospitalization. This leads to the conclusion that the greater the severity of the case is, the longer the hospitalization will be.

One confounding factor that the present study was unable to avoid was indirect favoring of the result from the group that did not have assistance from the center. There was the possibility that healthcare professionals might only have consulted the center once, in relation to one particular case, thus obtaining information on how to manage that case. Thereafter, they might have applied this information to similar cases, without activating the poison control center. The involvement of the poison control center was only measured in this study in terms of confirmations that requests had been made in relation to the cases in question.

The present study has limitations that have been pointed out previously. In particular, the source of the data for the cohort was non-computerized medical files; the study population was relatively small; and it was not possible to make a severity assessment on the entire patient group.

The results in this study were obtained from a public emergency service in a city in northern Brazil, the region of the country that has the lowest proportion of healthcare professionals per inhabitant.^34^ Furthermore, the sociodemographic characteristics of this municipality differ from those of other places.

One implication for further research that can be highlighted from the present study is that studies of high-quality design, with control over bias and confounding factors, need to be planned in order to achieve a better level of evidence. Prospective data and a larger sample size would reduce the uncertainty of the findings. Investigations on relevant outcomes, such as mortality, significant morbidity and cost-effectiveness, would enhance the validity of the conclusions.

## CONCLUSION

A statistically significant difference in length of hospital stay was found between poisoned patients who received assistance from a poison control center and those who did not obtain remote assistance, such that hospitalization was shorter among patients with assistance from the center. The poison control center may have reduced the length of hospitalization among the patients who received the intervention, and this result has implications relating to reduced morbidity due to poisoning and lower hospital treatment costs in such situations.
